# USING SOMATOSENSORY GAMES TO IMPROVE HEALTH AND SOCIAL ENGAGEMENT OF TAIWANESE OLDER ADULTS IN A COMMUNITY

**DOI:** 10.1093/geroni/igz038.3357

**Published:** 2019-11-08

**Authors:** Duan-Rung Chen, Ya-Mei Chen, Winston Tseng Tseng

**Affiliations:** 1 National Taiwan University, Taipei, Taiwan; 2 School of Public Health, California, United States

## Abstract

Objective. Playing games has become a new way to enhance the physical activity, quality of life, social engagement of older adults. This study aims to conduct a 6-month somatosensory game program, inviting older adults to play Microsoft Xbox Kinect games and study whether games can bring benefits to them. Methods. A total of 70 community-dwelling older adults (35 as experimental group, 35 as controls) were recruited. The experiment group played somatosensory games twice per week in a local health center. These games contained three types of categories: 1) Tournament games (for upper limb and lower limb); 2) Single games (for aerobic exercise and muscle training);3) Puzzle games (for collaboration and group dynamics): Results. After 6 months, in the experiment group, the body mass index decreased from 23.45 to 23.29 (p<0.03). In muscular endurance category, 30 second chair rise jumped from 18 to 23.07 (p<0.0001). And 2 min leg lifting increased from 119.48 to 137.75 (p<0.001). In the flexibility category, back scratch test from right hand on top and left hand on top both improved (p<0.026; p<0.46, respectively). Trend analysis indicates that the improvements in 30 second chair rise (p<0.0001) and 2 min leg lifting (p<0.001) is linear. The intensity of social interactions increased noticeably in the experiment group. The SF8 health survey also revealed that experimental group perceived lower level of bodily pain. No significant improvement in the control group in all categories. Conclusion. Playing somatosensory games regularly may help older adults physically and socially.

